# LRPPRC-Driven Oxidative Phosphorylation Is Associated with Elesclomol-Induced Cuproptosis in Ovarian Cancer

**DOI:** 10.3390/ijms27010451

**Published:** 2025-12-31

**Authors:** Ying Wu, Wenda Zhang, Shanshan Jiang, Sailong Liu, Jing Su, Liankun Sun

**Affiliations:** Department of Pathophysiology, College of Basic Medical Sciences, Jilin University, Changchun 130021, China; wuying22@mails.jlu.edu.cn (Y.W.); zhangwd24@mails.jlu.edu.cn (W.Z.); jiangss23@mails.jlu.edu.cn (S.J.); lsl24@mails.jlu.edu.cn (S.L.)

**Keywords:** cuproptosis, mitochondria, complex IV, ovarian cancer, LRPPRC

## Abstract

Mitochondrial oxidative phosphorylation serves as a critical driving force in the progression of ovarian cancer. Recent studies have demonstrated that copper induces mitochondrial-dependent programmed cell death by directly binding to the thioacylated components of the tricarboxylic acid (TCA) cycle. The involvement of copper in OXPHOS complex IV, a rate-limiting step in the mitochondrial respiratory chain, suggests that the role of mitochondria in mediating copper-induced cell death can be further elucidated through the study of OXPHOS complex IV. The findings of this study indicate that the cuproptosis process in ovarian cancer, induced by Elesclomol, is associated with mitochondrial complex IV, with LRPPRC identified as a crucial factor. Following Elesclomol treatment of ovarian cancer cells, there was a notable increase in mitochondrial reactive oxygen species (ROS), a significant accumulation of the copper death marker protein DLAT, and a marked decrease in the lipoic acid synthesis-related protein FDX1. Furthermore, the expression levels of copper ion transporters ATP7B and CTR1, which are involved in the assembly and translation of complex IV, as well as the core subunit MTCO1 of complex IV, the copper chaperone protein SCO1, and the interacting protein LRPPRC, were significantly diminished. Inhibition of the IV-stabilizing protein LRPPRC in the ovarian cancer cell lines A2780 and SKOV3 through RNA interference resulted in increased sensitivity to Elesclomol. Concurrently, the expression levels of FDX1, LIAS, LIPT1, SCO1, and MTCO1 decreased significantly. These findings suggest that LRPPRC plays a role in inhibiting the expression of lipoic acid and copper chaperone proteins during Elesclomol-induced copper death in ovarian cancer. This inhibition collectively diminishes the expression and activity changes in complex IV, induces mitochondrial dysfunction, and promotes cuproptosis in ovarian cancer. This study further demonstrates that inhibiting the oxidative phosphorylation complex IV can enhance copper-induced cell death in ovarian cancer.

## 1. Introduction

Ovarian cancer ranks among the most lethal gynecological malignancies [1]. Mitochondria serve as essential hubs for cellular metabolism and signal transduction [2]. It is well established that mitochondria influence the sensitivity of tumor cells to chemotherapy agents [3,4,5]. However, numerous issues concerning its mechanism of action remain to be elucidated. Previous studies have shown that the anti-cancer drug Elesclomol, which targets mitochondrial metabolism, frequently induces oxidative stress, DNA damage, and cell cycle arrest to kill tumor cells [6,7,8]. Recent studies have identified elesclomol-induced cuproptosis as a mitochondria-related form of cell death [9]. Elesclomol facilitates the catalytic reduction of ES-Cu (II) to Cu (I) by ferredoxin 1 (FDX1), subsequently releasing it into the mitochondria [10]. This compound also binds to the acylase involved in the tricarboxylic acid (TCA) cycle, promotes the aggregation of the protein DLAT, and induces protein toxic stress. Furthermore, it has been established that cuproptosis is contingent upon mitochondrial respiration [11]. OXPHOS complex IV serves as the rate-limiting step in the mitochondrial respiratory chain [12]. The key copper loss protein FDX1 provides electrons not only for lipoate synthase (LIAS) but also for the assembly of cytochrome C oxidase (complex IV) via the protein COX15 [13]. Additionally, FDX1 is essential for the synthesis of heme, which is required for the composition of lipoic acid and the IV subunit of the mitochondrial complex.

Complex IV, the last rate-limiting step in the respiratory chain and the regulatory center for oxidative phosphorylation [12], is considered a key molecule in determining cell fate. Complex IV is a copper-heme A heteromultimeric complex consisting of 3 catalytic core subunits MTCO1, MTCO2, MTCO3 encoded in mitochondrial DNA and 11 subunits encoded in the nuclear genome [14,15]. Recent studies have shown that FDX1 regulates expression of complex IV protein MTCO1 in rat cells and is considered essential for mitochondrial complex IV biogenesis in mammalian cells [16]. It indicates that the role of mitochondria in cuproptosis can be further elucidated from the perspective of mitochondrial complex IV.

Recent studies have found that LRPPRC is highly expressed in ovarian cancer tissues [17,18,19]. LRPPRC plays an important role in tumorigenesis, metastasis, and drug resistance by regulating cancer epigenetic modification, signal transduction, cancer metabolism, and cancer immunity. It has also been found to be negatively correlated with the overall survival and recurrence-free survival of ovarian cancer patients. Previous studies have shown that LRPPRC can not only stabilize and translate the mRNA of complex IV [20], thereby affecting oxidative phosphorylation in tumor cells [21,22,23]. Moreover, it has been found that the dysregulation of LRPPRC expression affects cancer progression through upstream regulators, interacting partners, and downstream targets [24]. The interacting partners include mitochondrial proteins such as COX10/14/15, cytochrome oxidase subunit 1(TACO1), SCO1/2, and SRA stem-loop-interacting RNA-binding protein (SLIRP), which play important roles in maintaining complex IV [25]. This suggests that taking LRPPRC as an entry point, detecting the expression of copper ion chaperone proteins can clarify the relationship between complex IV and cuproptosis from another perspective, providing new possibilities for increasing the sensitivity to chemotherapy drugs.

## 2. Results

### 2.1. Sensitivity to Cuproptosis in Ovarian Cancer Cells Is Associated with Mitochondrial Function

**Figure 1:**
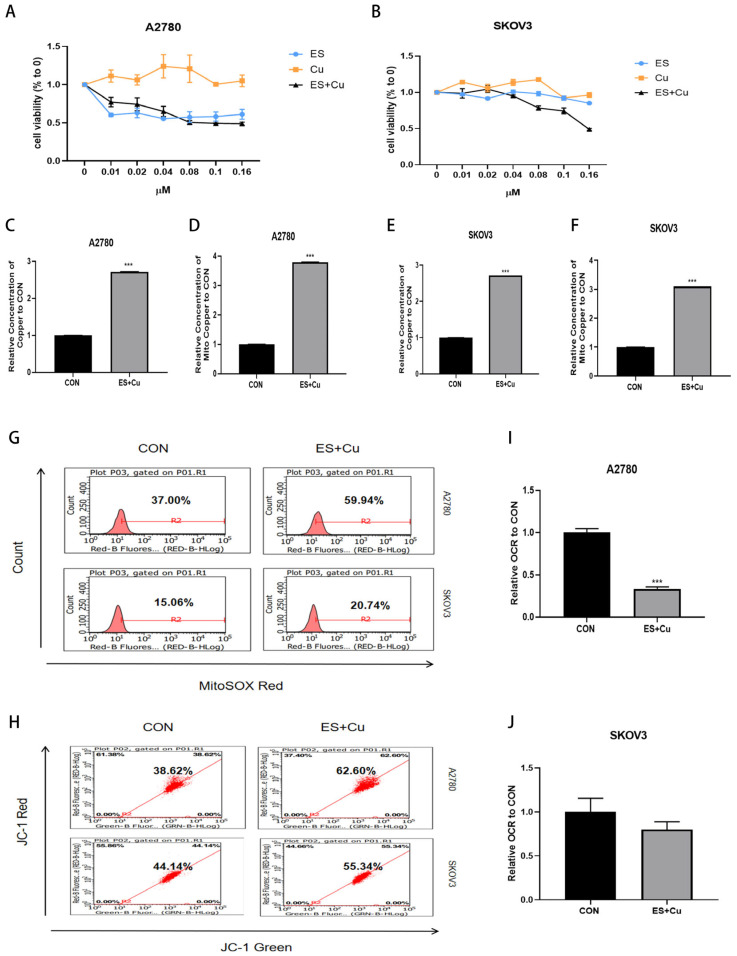
(**A**) Changes in the proliferation rates of A2780 cells and (**B**) SKOV3 cells after treatment with Elesclomol, CuCl_2_, and Elesclomol + CuCl_2_ (1:1) detected by the MTT assay; (**C**–**F**) Total and mitochondrial Cu^2+^ contents in A2780 and SKOV3 cells; (**G**) Changes in mitochondrial reactive oxygen species in A2780 and SKOV3 cells after treatment with Elesclomol + CuCl_2_ detected by the MitoSOX Red probe; (**H**) Changes in mitochondrial membrane potential in A2780 and SKOV3 cells after treatment with Elesclomol + CuCl_2_ detected by the JC—1 staining solution; (**I**,**J**) Changes in extracellular oxygen consumption rate in A2780 and SKOV3 cells after treatment with Elesclomol + CuCl_2_ detected by the OCR probe; Quantitative data represent the mean ± standard error of three independent experiments; *** *p* < 0.001 compared with the Control (CON) group.

Elesclomol was employed to assess the varying sensitivity of two ovarian cancer cell lines to cuproptosis. Specifically, SKOV3 cells exhibited reduced sensitivity to Elesclomol + CuCl_2_ (Figure 1A,B), with minimal impact on cell growth observed upon the addition of Elesclomol alone. The A2780 cells demonstrated ignificant drug sensitivity when treated with Elesclomol alone. In subsequent experiments, a combination of Elesclomol and CuCl_2_ (1:1) was employed. Furthermore, Elesclomol was found to enhance the Cu^2+^ content in both ovarian cancer cells and mitochondria during the induction of cuproptosis (Figure 1C–F). To elucidate the relationship between cuproptosis and mitochondrial function, we employed the MitoSOX-red probe and JC-1 staining solution to assess alterations in mitochondrial reactive oxygen species (ROS) and membrane potential. Additionally, we utilized the OCR probe to measure the extracellular oxygen consumption rate, thereby evaluating changes in mitochondrial function. Treatment with elesclomol resulted in an increase in mitochondrial ROS (Figure 1G), a decrease in membrane potential (Figure 1H), and a reduction in the extracellular oxygen consumption rate (Figure 1I,J) in the ovarian cancer cell lines A2780 and SKOV3, ultimately leading to a decline in mitochondrial function. A2780 cells exhibiting greater drug sensitivity demonstrated more pronounced alterations in mitochondrial function. These findings indicate that the sensitivity of ovarian cancer cells to copper-induced cell death is associated with mitochondrial function.

### 2.2. Elesclomol Promotes Ovarian Cancer Cuproptosis by Reducing the Activity of Complex IV

**Figure 2:**
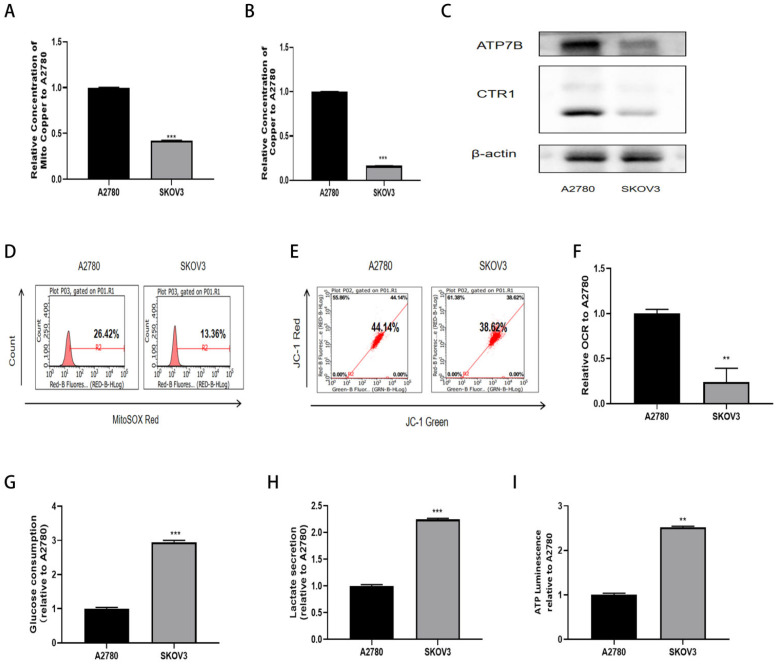
The mitochondrial (**A**) and overall (**B**) Cu^2+^ contents in A2780 and SKOV3 cells; (**C**) Western Blot was used to detect the expression differences of copper ion transporters in A2780 and SKOV3 cells; (**D**) MitoSOX Red probe was used to detect the differences in mitochondrial reactive oxygen species in A2780 and SKOV3 cells; (**E**) JC-1 staining solution was used to detect the differences in mitochondrial membrane potential in A2780 and SKOV3 cells; (**F**) OCR probe was used to detect oxygen consumption rate in A2780 and SKOV3 cells; (**G**–**I**) Biochemical kits were used to detect the differences in glucose uptake, lactate production and ATP production in A2780 and SKOV3 cells; Quantitative data represent the mean ± standard error of three independent experiments; ** *p* < 0.01, *** *p* < 0.001 compared with the A2780 group.

To elucidate the influence of copper metabolism on the differential sensitivity to Elesclomol, we first quantified the Cu^2+^ levels and assessed the expression of copper ion transporters in two distinct cell types. The findings indicated that the Cu^2+^ concentration in mitochondria (Figure 2A) and the total Cu^2+^ content (Figure 2B) were significantly lower in the drug-resistant SKOV3 cells. Additionally, the expression levels of copper ion transporters were also low expression (Figure 2C). These results confirm that sensitivity to cuproptosis correlates with both Cu^2+^ content and the high expression of copper ion transporters. Previous studies have demonstrated that cuproptosis is contingent upon mitochondrial respiration, with variations in energy generation modes and metabolism among tumor cells serving as significant factors influencing their drug sensitivity [11]. Additionally, a comparative analysis of mitochondrial functions indicated that SKOV3 cells exhibited reduced mitochondrial ROS expression and elevated membrane potential levels (Figure 2D,E). To further investigate the metabolic modes of the two cell types, we compared the basal cellular oxygen consumption rate (Figure 2F), glucose uptake (Figure 2G), and the production of lactate (Figure 2H) and ATP (Figure 2I). The results indicated that SKOV3 cells primarily relied on glycolysis, whereas A2780 cells utilized oxidative phosphorylation (OXPHOS) as their main metabolic pathway. These findings suggest that sensitivity to cuproptosis is associated with OXPHOS, with cells exhibiting active mitochondria demonstrating increased sensitivity to Elesclomol in ovarian cancer cells.

**Figure 3:**
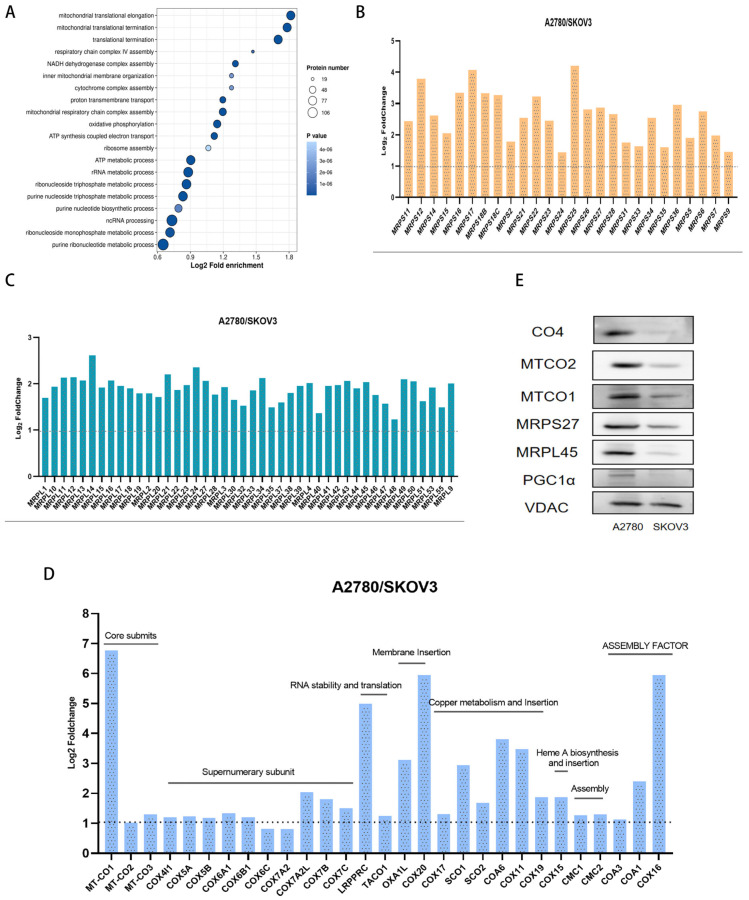
(**A**) Differential analysis of mitochondrial proteomics in A2780 and SKOV3 cells, and enrichment of downregulated biological processes in SKOV3 cells compared with A2780 cells. *p* values are all presented in scientific notation (4e-06 means 4 × 10^−6^); (**B**) Upregulated small ribosomal subunits in A2780 cells compared with SKOV3 cells; (**C**) Upregulated large ribosomal subunits in A2780 cells compared with SKOV3 cells; (**D**) Enrichment of proteins related to complex IV assembly, translation, and copper ion transport that are upregulated in A2780 cells compared with SKOV3 cells; (**E**) Western Blot detection of the mitochondria protein expression changes. The area above the dotted line represents the fold increase in gene expression of A2780 compared to SKOV3 cells in (**B**–**D**).

To elucidate the relationship between oxidative phosphorylation (OXPHOS) and cuproptosis, we conducted proteomic analyses on the mitochondria of two cell types. The findings indicated that proteins downregulated in SKOV3 cells, relative to A2780 cells, were predominantly associated with mitochondrial protein translation and the assembly of respiratory chain complex IV (Figure 3A). A further classification analysis of the proteins involved in the synthesis and assembly of complex IV in SKOV3 and A2780 cells revealed that A2780 cells, which exhibit greater sensitivity to Elesclomol, showed elevated expression of mitochondrial-encoded core subunits of complex IV (MTCO1, MTCO2, MTCO3), nuclear-encoded subunits (COX4i1, COX5A, COX5B, COX6A1, COX6B1, COX6C, COX7B, COX7C), proteins that promote RNA stability and translation (LRPPRC, TACO1), inner membrane insertion proteins (OXA1L, COX20), proteins associated with copper metabolism (COX17, SCO1, SCO2, COA6, COX11, COX19), proteins involved in complex assembly (CMC1, CMC2), and ribosomal subunits necessary for mitochondrial protein translation (Figure 3B–D). This finding indicates that A2780 cells, which exhibit high OXPHOS expression and greater sensitivity to Elesclomol, have elevated levels of mitochondrial complex IV compared to SKOV3 cells, which display low OXPHOS expression and reduced sensitivity. To further validate these proteomic results, we conducted Western Blot experiments (Figure 3E).

**Figure 4:**
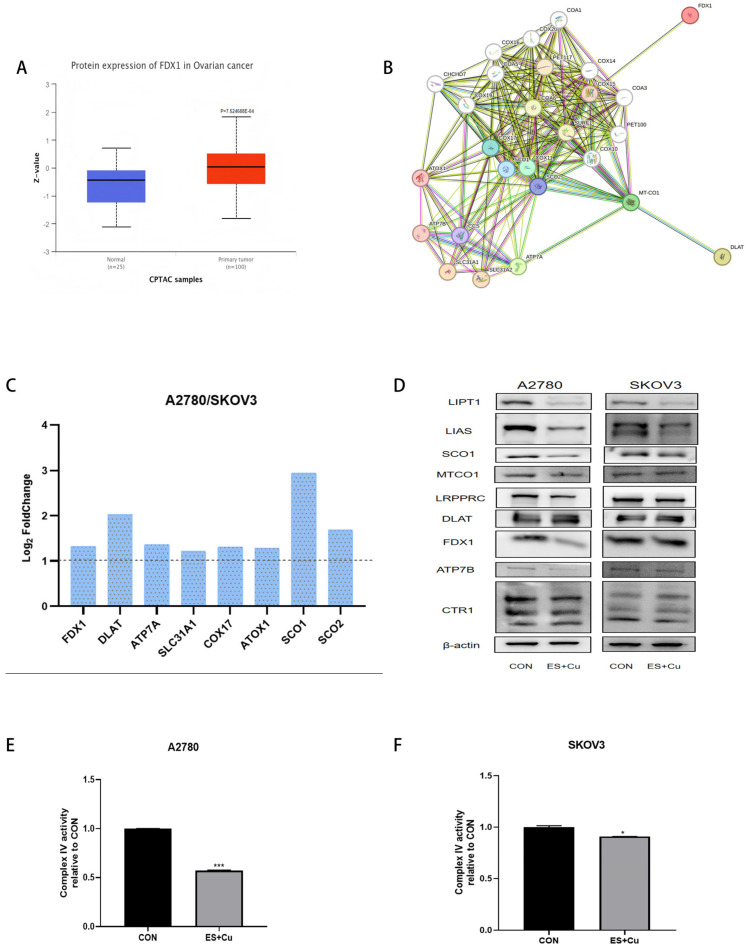
(**A**) Analysis of the difference in FDX1 protein expression between normal and ovarian cancer tissues using the UALCAN website; (**B**) Analysis of the PPI between FDX1 and complex IV using the STRING database; (**C**) The levels of proteins related to cuproptosis extracted from isolated mitochondria upregulate in A2780 cells compared with SKOV3 cells. The area above the dotted line represents the fold increase in gene expression of A2780 compared to SKOV3 cells; (**D**) Western Blot detection of the whole-cell expression changes in the death-related protein LRPPRC, MTCO1, FDX1, lipoic acid synthesis proteins LIPT1 and LIAS, and copper ion transport proteins ATP7B and CTR1 (SLC31A1) in A2780 and SKOV3 cells after treatment with Elesclomol + CuCl_2_ (1:1). (**E**,**F**) After reatment with 0.1 μM Elesclomol + CuCl_2_ (1:1), the activity of complex IV were detected in A2780 and SKOV3 cells. * *p* < 0.05, *** *p* < 0.001 compared with the CON(Control) group.

To investigate the relationship between cuproptosis and complex IV in ovarian cancer, we initially employed the online analysis platform UALCAN (https://ualcan.path.uab.edu/index.html, accessed on 7 October 2025), which revealed that cuproptosis marker protein FDX1 is significantly overexpressed in ovarian cancer tissues (Figure 4A) [16]. Additionally, FDX1 serves as an upstream regulator of protein lipoylation and is implicated in cell death triggered by the copper ionophore Elesclomol. Analysis using the String database indicated that FDX1 is associated with COX15, a protein involved in the synthesis of heme A within complex IV (Figure 4B). Previous studies have indicated that the overexpression of heme A synthase COX15 partially restored the abundance of MTCO1 protein following FDX1 knockout in rat cardiomyocytes. A comparative analysis of mitochondrial proteomics between ovarian cancer SKOV3 cells and A2780 cells demonstrated that FDX1, the copper transporter SLC31A1 (CTR1), ATP7A, and the copper chaperone proteins SCO1, SCO2, and antioxidant 1 copper chaperone (ATOX1), which are associated with complex IV, were all significantly upregulated in A2780 cells (Figure 4C). We subsequently extracted proteins from A2780 and SKOV3 ovarian cancer cells following elesclomol-induced cuproptosis. The experimental results indicated that, after treatment with elesclomol, DLAT levels increased, while the expression of FDX1, lipoic acid synthesis-related proteins LIPT1 and LIAS, copper ion transporters ATP7B and CTR1, the core subunit MTCO1 of complex IV, the stabilizing protein LRPPRC, and the copper chaperone protein SCO1 decreased. The decrease was more pronounced in A2780 cells, which exhibit heightened sensitivity to cuproptosis (Figure 4D). The activity changes in complex IV were assessed using biochemical kits. The results indicated that Elesclomol could diminish the activity of complex IV (Figure 4E,F). These findings suggest that the activity of complex IV may contribute significantly to the enhanced sensitivity to cuproptosis observed in A2780 cells. Our findings indicate that the sensitivity of ovarian cancer cells to cuproptosis is contingent upon the expression of proteins associated with Complex IV. Furthermore, this study demonstrates that, during elesclomol-induced copper overload, the cell death process is regulated not only by FDX1 but also by the essential assembly factors of Complex IV, LRPPRC and SCO1.

### 2.3. Mechanism by Which LRPPRC Affects Elesclomol Sensitivity Through Complex IV

**Figure 5:**
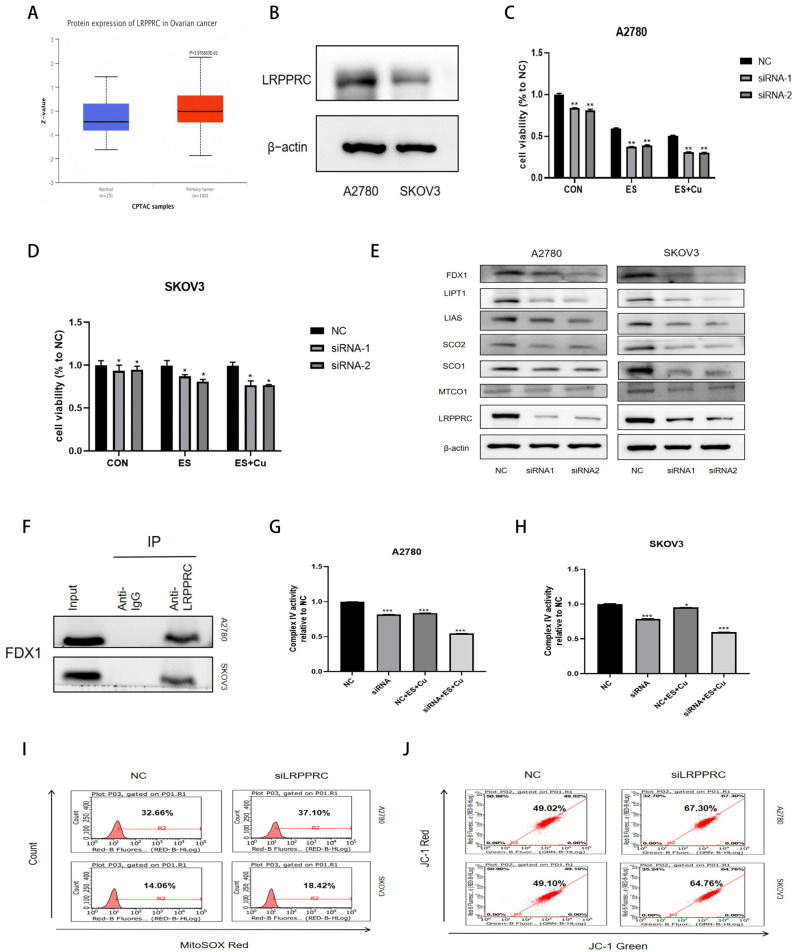
(**A**) Analysis of the protein expression difference in LRPPRC between normal and ovarian cancer tissues on the UALCAN website (http://ualcan.path.uab.edu/index.html, accessed on 7 October 2025); (**B**) Detection of the expression difference in LRPPRC in A2780 and SKOV3 cells by Western Blot; After knocking down LRPPRC in A2780 and SKOV3 cells, the MTT method was used to detect the changes in the proliferation rates of A2780 cells (**C**) after treatment with 0.04 μM Elesclomol, and 0.04 μM Elesclomol + CuCl_2_ (1:1); SKOV3 cells (**D**) after treatment with 0.1 μM Elesclomol, and 0.1 μM Elesclomol + CuCl_2_ (1:1); (**E**) Detection of the expression changes in cuproptosis-related protein FDX1, lipoic acid synthesis proteins LIPT1 and LIAS, and complex IV assembly proteins SCO1 and SCO2 after knocking down LRPPRC in A2780 and SKOV3 cells; (**F**) A2780 and SKOV3 cell lysates were incubated with anti-LRPPRC antibody or IgG antibody (negative control), and conjugated to the antibody were immunoprecipitated (IP). Captured protein was detected by Western Blo using FDX1 specific antibody; (**G**) The activity of complex IV were detected in A2780 cells after knocking down the expression of LRPPRC and 0.04 μM Elesclomol + CuCl2 (1:1); (**H**) The activity of complex IV were detected in SKOV3 cells after knocking down the expression of LRPPRC treatment with 0.1 μM Elesclomol + CuCl2 (1:1); (**I**) Detection of the changes in mitochondrial reactive oxygen species in A2780 and SKOV3 cells after knocking down LRPPRC using the MitoSOX Red probe; (**J**) Detection of the changes in mitochondrial membrane potential in A2780 and SKOV3 cells after knocking down LRPPRC using JC-1 staining solution; Quantitative data represent the mean ± standard error of three independent experiments; * *p* < 0.05, ** *p* < 0.01, *** *p* < 0.001 compared with the NC (Negative Control) group.

We initially assessed the expression of LRPPRC, a protein that stabilizes complex IV mRNA and facilitates protein translation, in ovarian cancer tissues (Figure 5A). The results revealed that LRPPRC was significantly upregulated in these tissues, indicating its potential as a target for inducing cuproptosis in ovarian cancer. Additionally, we compared LRPPRC expression levels in ovarian cancer cell lines (Figure 5B). The findings demonstrated that LRPPRC was markedly elevated in A2780 cells, which also exhibited high levels of FDX1 and MTCO1. Following the knockdown of LRPPRC, we observed an increased sensitivity to Elesclomol (Figure 5C,D), an enhancement in mitochondrial reactive oxygen species production (Figure 5I), and a reduction in membrane potential (Figure 5J), all of which adversely affected mitochondrial function. These results suggest that LRPPRC plays a critical role in the cuproptosis process induced by Elesclomol.

In A2780 cells exhibiting high levels of LRPPRC expression, treatment with Elesclomol resulted in a significant reduction in LRPPRC levels. To investigate the role of LRPPRC in Elesclomol-induced cuproptosis through the regulation of complex IV, additional knockdown of LRPPRC was conducted. This intervention revealed a decrease in the expression of FDX1, LIAS, and LIPT1, indicating that LRPPRC may function as an upstream regulator of FDX1 (Figure 5E). Next, immunoprecipitation was employed using LRPPRC antibody for pull-down to identify FDX1 bound to LRPPRC (Figure 5F). LRPPRC is essential for maintaining the stability of messenger RNA (mRNA) within mitochondria, including those that encode SCO1 and SCO2. Following the knockdown of LRPPRC in two ovarian cancer cell types (Figure 5E), we observed a reduction in the expression of chaperone proteins SCO1 and SCO2, with a more pronounced decrease in SCO1. Subsequently, the alterations in complex IV activity were assessed. The knockdown of LRPPRC significantly exacerbated the reduction in complex IV activity induced by Elesclomol (Figure 5G,H). This finding suggests that SCO1 plays a role in regulating the stability of complex IV in ovarian cancer cells. Knockdown of LRPPRC in ovarian cancer cells is believed to impact the assembly of complex IV by decreasing SCO1, while simultaneously affecting the biosynthesis of complex IV through the downregulation of FDX1 and lipoic acidylated proteins. This dual mechanism ultimately enhances the sensitivity of ovarian cancer cells to Elesclomol-induced cuproptosis.

## 3. Discussion

Mitochondrial dysfunction is closely linked to ovarian cancer [26]. Mitochondria function as a central hub for various forms of cell death [27]. Previous studies have investigated the enhancement of apoptosis, ferroptosis, and other cell death modalities in ovarian cancer cells as strategies to target mitochondria and increase the sensitivity of chemotherapy drugs used for ovarian cancer [28,29,30]. Recently, with the advancement of research on copper ion metabolism, mitochondria have emerged as significant contributors to cuproptosis. Zou et al. [31] demonstrated that inducing cuproptosis in ovarian cancer could inhibit tumor progression and augment the toxicity of cisplatin [32]. An increasing body of research indicates that, compared to healthy individuals, cancer patients exhibit significantly elevated copper levels in both tumor tissues and sera. These elevated levels are correlated with the etiology, severity, and progression of cancer. Targeting copper metabolism may represent a potential strategy for inhibiting ovarian cancer. In prostate cancer, elevated levels of CTR1, ATP7A, and ATP7B have been observed. Similarly, breast cancer is characterized by the overexpression of CTR1, ATP7B, ATOX1, and COX17. Copper overload can activate the Fenton reaction, instigated by copper ions, resulting in the production of harmful reactive oxygen species (ROS) and inducing oxidative stress damage in cells. In the context of cuproptosis, FDX1 serves as the direct target of elesclomol, which facilitates a distinct form of cuproptosis [33,34]. FDX1 regulates cellular protein lipoylation through direct binding to LIAS [35]. Moreover, the absence of FDX1 leads to mild copper deficiency in cells, potentially resulting in defects in the biogenesis of cytochrome C oxidase (complex IV), as copper is crucial for the stability of its copper-containing subunits. Given that cuproptosis is dependent on mitochondrial respiration, we compared the mitochondrial proteomes of two ovarian cancer cell types. This analysis revealed that sensitivity to cuproptosis correlates with the expression of the core Complex IV subunit MT-CO1 and its chaperone SCO1. Consistent with this finding, A2780 cells, which exhibit greater sensitivity to elesclomol than SKOV3 cells, express higher levels of FDX1, copper transporters (ATP7B and CTR1), and Complex IV proteins. We conducted a comprehensive analysis of the relationship between molecules involved in the synthesis of mitochondrial complex IV and cuproptosis.

Mitochondrial respiratory chain complex IV requires copper and heme A as essential cofactors for its assembly and functions, which include energy generation and substance metabolism. Copper chaperone proteins, such as COX11, COX17, COX19, COX23, SCO1, and SCO2, facilitate the transport of copper to cytochrome c oxidase (COX) [36]. These chaperone proteins play critical roles not only in copper ion transport but also in the assembly and synthesis of complex IV. Previous studies have demonstrated that treatment of the human erythroleukemia cell line K562 with the copper ion chelator bathocuproine disulfonate (BCS) results in reduced activity and expression of complex IV [37]. Additionally, non-cytotoxic copper overload can enhance mitochondrial energy metabolism in K562 cells and increase the expression of complex IV [38]. In this study, we observed that the expression of complex IV was downregulated following Elesclomol-induced copper overload, which resulted in the death of ovarian cancer cells. This phenomenon may be associated with the elevated expression of FDX1 in tumor tissues. One possible explanation is that the Fenton reaction, which occurs in the presence of excess copper, generates hydroxyl radicals (•OH). The impairment of respiratory chain function, particularly the inhibition of complex IV, leads to increased electron leakage and the production of superoxide anions (O_2_•^−^). These superoxide anions can disproportionate to hydrogen peroxide (H_2_O_2_), thereby providing additional substrates for the Fenton reaction and establishing a vicious cycle of oxidative stress. Furthermore, FDX1 is consumed during cuproptosis, resulting in decreased expression that fails to satisfy the requirements of complex IV for copper ions and heme A. Additionally, excess “free” or “unbound” copper ions, which exist in a non-protein-bound state, may be incorrectly incorporated into the copper sites of complex IV. This misincorporation can alter the spatial conformation of the enzyme, thereby obstructing normal electron transfer and oxygen reduction reactions. Consequently, further analysis and exploration are warranted based on the metabolic characteristics of normal and tumor cells.

LRPPRC is encoded by the LRPPRC/LRP130/GP130/LSFC gene and plays a significant role in tumorigenesis, metastasis, and drug resistance by regulating cancer epigenetic modifications, signal transduction, cancer metabolism, and cancer immunity [24,39]. In addition to its involvement with copper, LRPPRC, which contributes to the stability and active expression of complex IV mRNA, is highly expressed in various tumor tissues, including ovarian cancer [22], breast cancer [40], lung cancer [41], and gastric cancer [42]. Targeting LRPPRC in the context of oxidative phosphorylation may inhibit the progression of ovarian cancer. No studies have yet reported the relationship between LRPPRC and cuproptosis, or its key molecule FDX1, in ovarian cancer. This study demonstrates that A2780 cells, which are sensitive to Elesclomol, exhibit higher LRPPRC expression compared to SKOV3 cells, which are resistant to Elesclomol. Following treatment with Elesclomol, LRPPRC expression decreased in both A2780 and SKOV3 cells, with a more pronounced reduction observed in A2780 cells. This finding suggests that LRPPRC plays a role in the Elesclomol-induced cuproptosis process. Furthermore, it was found that inhibiting LRPPRC expression not only diminishes the translation of the core subunit MTCO1 of complex IV and the copper chaperone molecule SCO1 in A2780 and SKOV3 ovarian cancer cells, but also reduces the expression levels of FDX1 and LIAS. However, the observation that SCO1 was minimally affected in A2780 cells, which are sensitive to Elesclomol, while it was significantly impacted in the non-sensitive SKOV3 cells. The possible reason could be the basal expression levels of LRPPRC and SCO1 differ between the ovarian cancer cell lines A2780 and SKOV3, with SKOV3 exhibiting lower expression levels (as shown in Figure 3D). When LRPPRC is knocked down, the translation of the core subunit of complex IV is exacerbated, further impairing the assembly of complex IV. This disruption triggers cellular adaptive mechanisms, such as the mitochondrial unfolded protein response (UPR^mt^) [43], which actively down-regulates the already low levels of the nuclear-encoded SCO1 protein to prevent cytotoxicity resulting from protein accumulation. UPR^mt^ is closely associated with chemotherapy resistance in tumor cells [44], which may explain the significant decrease in SCO1 observed in SKOV3 cells. In alignment with these findings, we present new evidence that LRPPRC plays a role in promoting drug resistance in ovarian cancer cells through the regulation of the cuproptosis pathway. LRPPRC not only downregulates FDX1, thereby affecting the synthesis of LIPT1/LIAS, but also jointly regulates the biosynthesis of complex IV subunits by influencing the copper chaperone protein SCO1. This further elucidates the relationship between mitochondrial complex IV and cuproptosis in ovarian cancer cells.

## 4. Materials and Methods

### 4.1. Cell Lines and Cell Culture

A2780 (SCSP-5477) and SKOV3 (SCSP-5214) were purchased from the Shanghai Cell Bank of Chinese Academy of Sciences (Shanghai, China). Cells were grown in RPMI-1640 (Bio-Channel, Nanjing, China) supplemented with 10% fetal bovine serum (Bio-Channel, China) at 37 °C in 5% CO_2_.

### 4.2. Reagents and Antibodies

Methylthiazolyldiphenyl-tetrazolium bromide (MTT) (S6821) and Elesclomol (S1052) were commercially sourced from Selleck (Houston, TX, USA). Anti-CTR1 (T510261, Abmart, Shanghai, China), anti-ATP7B (19786-1-AP, Proteintech, Wuhan, China), anti-MTCO1 (A17889, ABclonal, Wuhan, China),anti-MTCO2 (A17965, ABclonal, Wuhan, China), anti-Actin (HRP-60008, Proteintech, Wuhan, China), anti-COX4 (11242-1-AP, Proteintech, Wuhan, China), anti-VDAC (FNab09385, Wuhan, China), anti-MRPS27 (17280-1-AP, Proteintech, Wuhan, China), anti-MRPL45 (PTM-5866, PTMBIO, Hangzhou, China), anti-PGC1α (66369-1-Ig, Proteintech, Wuhan, China), anti-FDX1 (12592-1-AP, Proteintech, Wuhan, China), anti-DLAT (13426-1-AP, Proteintech, Wuhan, China), anti-LIAS (HA722717, HUABIO, Hangzhou, China), anti-SCO1 (12614-1-AP, Proteintech, Wuhan, China), anti-SCO2 (21223-1-AP, Proteintech, Wuhan, China), anti-LRPPRC (PTM-5856, PTMBIO, Hangzhou, China).

### 4.3. Cell Viability Assay

Cells were seeded in 96-well plates overnight at a density of 8000 cells/well. Then, the cells were treated with various reagents for 24 h. Cell viabilities were assessed by MTT assay, and absorbance values were measured at 490 nm using a Vmax Microplate Reader (Molecular Devices, Sunnyvale, CA, USA).

### 4.4. Flow Cytometry Analysis

Cells were seeded in 6-well plates overnight at a density of 3 × 10^5^ cells/well. Then, the cells were exposed to various reagents for the indicated times. JC-1 (Beyotime Biotechnology, Shanghai, China) was used to evaluate the mitochondrial membrane potential. The production of mtROS was evaluated using Mitosox (Thermo Fisher Scientific, Waltham, MA, USA). The levels were quantified using Guava Easycyte flow cytometry (Guava easycyte, Hayward, CA, USA).

### 4.5. Gene Knockdown

siRNAs targeting LRPPRC and Negative Control siRNAs were purchased from IBSBIO (Shanghai, China). Cells were seeded in 6-well plates for 24 h prior to transfection with siRNA targeting RAGE or corresponding non-targeting controls. A total of 2.0 μg of siRNA was transfected in OptiMem medium (Gibco, Waltham, MA, USA) using TurboFect Transfection Reagent (Thermo Scientific, Waltham, MA, USA). At 24 h post-transfection, the cells were harvested and assayed for RNA and protein expression levels of the target of interest. The siRNA targeting sequences were as follows: LRPPRC siRNA1(CCUCAAAGGAAUGCAAGAAUUTT); siRNA2(CGCAGCUUUAAGAGGUGAAAUTT).

### 4.6. Cell Fractionation

Cells were lysed in 1% Triton X-100 phosphate-buffered saline (PBS) with protease inhibitors on ice for 30 min and then centrifuged for 30 min at 16,000× *g*. The insoluble fraction was lysed in 2% SDS with protease inhibitors at 60 °C for 1 h, followed by centrifugation for 30 min at 16,000× *g*. The resulting supernatants and pellets were recovered as the soluble and insoluble fractions, respectively. Proteins were resolved by SDS–PAGE in 1 × Laemmli buffer and visualized by Coomassie staining and immunoblotting.

### 4.7. Mitochondria Isolation

Mitochondria extraction was conducted according to the instructions of the Mitochondria Isolation kit (Invent Biotechnologies, Eden Prairie, MN, USA). Briefly, 1 × 10^7^ cells were harvested by low-speed centrifugation (500× *g*, 5 min). The cells were washed twice with cold PBS, before resuspending the cell pellets in 250 μL buffer A. The cell suspensions were incubated on ice for 10 min, before transferring to a filter cartridge and centrifuging at 14,000 rpm for 30 s. After discarding the filter, the pellets were resuspended and centrifuged at 3000 rpm for 1 min (the nucleus was precipitated). Subsequently, the supernatants were transferred to fresh 2.0 mL tube, before adding 400 μL buffer B to the tube. The pellets were resuspended and centrifuged at 14,000 rpm for 10 min, before removing the supernatant containing cytoplasmic and plasma membrane proteins. The pellets were resuspended in 200 μL buffer B and centrifuged at 10,000 rpm for 5 min (the precipitate contained organelles larger than mitochondria). The supernatants were transferred to a fresh 2.0-mL tube, 1.5 mL cold PBS was added to the tube and centrifuged it at 16,000 rpm for 15 min. The supernatant contained organelles smaller than mitochondria, while the pellets contained isolated mitochondria.

### 4.8. Differentially Expressed Mitochondrial Protein and Its Functional Enrichment Analyses

The ratio of the mean relative quantitative values of proteins in the two groups of samples was taken as the fold change (FC). The relative quantitative values of proteins in the two groups of samples were tested by *t*-test, and the corresponding *p*-values were calculated. When the *p*-value was <0.05, the change in differential expression level was >1.5 as the significantly up-regulated change threshold, and <1/1.5 as the significantly down-regulated change threshold. Functional terms with fold enrichment > 1.5 and *p*-value < 0.05 were considered significant.

### 4.9. Western Blotting

Whole-cell lysates were prepared and quantified according to standard protocols. Lysates were diluted in 5 × SDS-PAGE loading buffer, boiled at 95 °C for 10 min, separated by SDS-PAGE, and then electrophoretically transferred to polyvinylidene difluoride membranes. The membranes were blocked with 5% milk followed by successive incubation with primary antibodies and peroxidase-conjugated secondary antibodies. The bands were visualized using Pierce ECL Western Blot Substrate (Thermo Scientific, Waltham, MA, USA).

Mitochondrial protein extraction was performed using the Beyotime cell mitochondria isolation kit (Beyotime, Beijing, China) according to manufacturer’s instructions. The isolated mitochondrial protein samples were subsequently subjected to Western blot analysis to determine the expression levels of specific mitochondrial proteins.

### 4.10. Complex IV Activity Assay

Mitochondrial complex IV activity was detected according to the Mitochondrial Respiratory Chain Complex IV Activity Assay Kit (Mlbio, Shanghai, China). After extracting the mitochondria, the mitochondria were resuspended and precipitated with 200 μL of Ragent II and 2 μL of Ragent III, and 20 μL of sample and 200 μL of working solution added, and the initial absorbance at 550 nm (0 min) and the absorbance after 37 °C (30 min) were measured, and the value of ΔA = A1 − A2 was calculated. Complex IV activity = 19.20 × ΔA ÷ Cpr.

### 4.11. Immunoprecipitation (IP)

Cells were lysed in cell lysis buffer NP40 containing protease inhibitor and allowed to incubate for 30 min at 4 °C. The protein samples were divided into IP and Input IgG groups. IgG antibodies (Proteintech, B900620) and LRPPRC antibodies (PTM-5856)were added to the IgG group and the IP group. Lysates containing 2 mg of total proteins were used to perform immunoprecipitation with 30 µL beads (Sigma, Merck KGaA, Darmstadt, Germany) for 30 min at 4 °C. Wash the magnetic beads with PBS and lyse the samples with RIPA lysis buffer containing protease inhibitors.

### 4.12. Statistical Analysis

Statistical analysis was performed using GraphPad Prism software (GraphPad Software 8.0.1, San Diego, CA, USA). Student’s *t*-test was used unless otherwise stated. SEM was used as the measure of variation from the population mean in all experiments. The degree of statistical significance was represented using asterisks: *p* ≤ 0.05 (*), *p* ≤ 0.01 (**) and *p* ≤ 0.001 (***).

## Data Availability

The original contributions presented in this study are included in the article. Further inquiries can be directed to the corresponding authors.
